# Burden of de novo mutations and inherited rare single nucleotide variants in children with sensory processing dysfunction

**DOI:** 10.1186/s12920-018-0362-x

**Published:** 2018-05-25

**Authors:** Elysa Jill Marco, Anne Brandes Aitken, Vishnu Prakas Nair, Gilberto da Gente, Molly Rae Gerdes, Leyla Bologlu, Sean Thomas, Elliott H. Sherr

**Affiliations:** 1 Department of Neurology, University of California, San Francisco, 675 Nelson Rising Lane, San Francisco, CA 9415 USA; 2 Department of Psychiatry, University of California, San Francisco, 401 Parnassus Ave, San Francisco, CA 94143 USA; 3 Department of Pediatrics, University of California, San Francisco, 550 16th Street, Box 0110, San Francisco, CA 94143 USA; 4 Department of Biostatistics & Epidemiology, University of California, San Francisco, 550 16th Street, 2nd Floor, Box #0560, San Francisco, CA 94158-2549 USA; 5 Institute of Human Genetics, University of California, San Francisco, 513 Parnassus Avenue, S965, San Francisco, CA 94143-0794 USA; 6 San Francisco, CA USA

**Keywords:** Sensory Processing Disorder, Autism, Neurodevelopment, Genetics, MBD5, FMN2

## Abstract

**Background:**

In children with sensory processing dysfunction (SPD), who do not meet criteria for autism spectrum disorder (ASD) or intellectual disability, the contribution of de novo pathogenic mutation in neurodevelopmental genes is unknown and in need of investigation. We hypothesize that children with SPD may have pathogenic variants in genes that have been identified as causing other neurodevelopmental disorders including ASD. This genetic information may provide important insight into the etiology of sensory processing dysfunction and guide clinical evaluation and care.

**Methods:**

Eleven community-recruited trios (children with isolated SPD and both biological parents) underwent WES to identify candidate de novo variants and inherited rare single nucleotide variants (rSNV) in genes previously associated with ASD. Gene enrichment in these children and their parents for transmitted and non-transmitted mutation burden was calculated. A comparison analysis to assess for enriched rSNV burden was then performed in 2377 children with ASD and their families from the Simons Simplex Collection.

**Results:**

Of the children with SPD, 2/11 (18%), were identified as having a de novo loss of function or missense mutation in genes previously reported as causative for neurodevelopmental disorders (MBD5 and FMN2). We also found that the parents of children with SPD have significant enrichment of pathogenic rSNV burden in high-risk ASD candidate genes that are inherited by their affected children. Using the same approach, we confirmed enrichment of rSNV burden in a large cohort of children with autism and their parents but not unaffected siblings.

**Conclusions:**

Our findings suggest that SPD, like autism, has a genetic basis that includes both de novo single gene mutations as well as an accumulated burden of rare inherited variants from their parents.

**Electronic supplementary material:**

The online version of this article (10.1186/s12920-018-0362-x) contains supplementary material, which is available to authorized users.

## Background

Sensory processing dysfunction (SPD) affects 5–16% of children and can contribute to long-term impairments in cognition, social development, and family well-being [1–3]. Additionally, hyper and hypo-sensitivity to sound and touch has recently been added to the symptom cluster for Autism Spectrum Disorders (ASD) in the most recent Diagnostic and Statistical Manual, DSM-5 [4]. We have recently shown that children with SPD, who do not meet criteria for ASD, have measurable differences in white matter microstructure predominantly in the posterior brain regions, which are critical to sensory perception and processing [5]. We have further demonstrated overlap between these brain findings in children with SPD and children with ASD, suggesting that there is not only a phenotypic overlap between SPD and ASD, but that there may be a mechanistic connection as well [6]. However, in our study, children with ASD have broader neural disruption, including key white matter tracts that subserve language, emotional memory, and processing. Approaches to address ASD mechanisms have included deep genetic analyses, including whole exome sequencing (WES), demonstrating that loss of function de novo mutations occur in genes that play important roles in neurodevelopmental pathophysiology. This type of rigorous approach to investigate the genetics of SPD remains to be undertaken, despite the growing recognition that SPD can present both as an isolated neurodevelopmental concern as well as a co-morbid condition for children who meet criteria for other behaviorally described conditions in the DSM-5 such as ASD, attention deficit disorders, and anxiety [7–10].

There are, however, some suggestions as to the genetic architecture of SPD. Numerous genetically mediated neurodevelopmental disorders have been reported to show increased sensory sensitivity, including triplet repeat disorders (e.g. Fragile X), chromosomal copy number variations (e.g. Williams syndrome), and single gene disorders (e.g. ARHGEF9) [11–13]. In addition, large population-based twin studies suggest that sensory over-responsivity (SOR) shows moderate heritability across sensory domain with 38% of auditory SOR and 52% of tactile SOR attributed to genetic factors [14].

The search for genes that explain the observed heritability in cognitive and behavioral disorders has been challenging. Despite that, recent WES with large ASD cohorts have shown that loss of function mutations in certain genes occur in 10–15% of patients with a frequency that provides strong statistical evidence for causality in ASD [15]. There is also evidence that ASD patients carry an oligogenic or polygenic combination of variations in “high-risk” neurodevelopment genes, each variant making either a large or small contribution to the phenotype [16, 17]. In fact, it is now posited that as many as 1000 genes can confer risk for ASD [18].

WES technology allows for the investigation of both de novo mutations and inherited risk polymorphisms when sequencing is performed for the index patient and his/her parents. In cohorts of children with intellectual disability or global developmental delay, the diagnostic rate using WES for de novo single gene etiologies was estimated to be 33% [19]. In an initial study of 238 families where only one member has autism, Sanders, et al. 2012 identified 16 loss of function mutations in probands, including nonsense, splice site and frame shift mutations [20]. In a follow up study, integrating both copy number variations and de novo loss of function mutations from WES, Sanders, et al. 2015 report 65 high-risk autism genes which show enrichment in protein-protein interactions and suggest two main sub-networks: chromatin regulation and synaptic control [21]. In this preliminary study, we sought to identify de novo loss of function mutations in children who presented with SPD to investigate monogenic etiologies. We further aimed to test whether there is an increased burden of inherited (or transmitted) rare Single Nucleotide Variants (rSNV) in high- and moderate- risk ASD genes when compared to non-transmitted rare variants in both our preliminary SPD cohort and in the larger ASD family cohort from the Simons Simplex Collection. We hypothesize that, as there are phenotypic and brain structure similarities in children with ASD or SPD, there may also be an overlap in genetic etiologies.

## Methods

This genetic cohort study aims to establish the occurrence of de novo missense and loss of function mutations in children with community diagnosed SPD. We further aimed to determine if there is a higher burden of transmitted rSNV in children with SPD and their parents in high and moderate-risk genes associated with ASD.

### Characteristics of participants

#### SPD cohort

We recruited 11 children (7 boys and 4 girls) with SPD and their biologic mother and father. Children were recruited from our existing Sensory Neurodevelopment and Autism Program (SNAP) cohort for whom we have neuroimaging, cognitive, and sensory processing characterization (see Table 1 for demographics). Informed consent was obtained from participants and parents, with assent of all participants from 12 to 18 years of age in accordance with the UCSF Institutional Review Board protocol. Inclusion criteria consist of a “Sensory Processing Disorder” diagnosis made by a community occupational therapist and a score on the Sensory Profile in the “Definite Difference” range (< 2% probability in a typically developing cohort) in one or more of the sensory domains (auditory, visual, oral/olfactory, tactile, vestibular, or multisensory processing). The Sensory Profile (Dunn, 1999) is a parent-report questionnaire that characterizes sensory experiences, behavior, and their functional impact. The domain scores were collectively used for differentiation of SPD and typically developing children. Higher scores indicate greater dysfunction. Subjects were excluded if they met research criteria for ASD which begins with screening using the parent report measure, the Social Communication Questionnaire (SCQ- ASD cut-off at 15), and confirmed using the direct assessment measure, the Autism Diagnostic Observation Schedule (ADOS); if they had cognitive impairment as defined as a full scale or performance IQ less than 70; or if they had a brain malformation on MRI, history of stroke or encephalitis, head injury with loss of consciousness > 15 min, multiple sclerosis, movement disorders, psychiatric disorders (e.g. bipolar disorder or schizophrenia), current history of pacemaker, ferromagnetic matter in body, claustrophobia or significant medical illness, premature delivery (gestational age < 36 weeks), or previously diagnosed genetic etiology for their neurodevelopmental condition.

**Table 1:** Probands demographics

	SPD cohort +/− Standard deviation [range]
Age	9.8 years +/− 1.3 [8–11]
VCI	121.5 +/− 11.6 [100–138]
PRI	111.5 +/− 16.7 [79–131]
SSP Total	116.4 +/− 18.0 [95–145]
Ethnicity	
Caucasian	8
Hispanic	0
Asian	0
Multiracial/Other	2
Unknown/Declined	1

*VCI* Verbal Comprehension Index of the Wechsler Intelligence Scale for Children-IV (WISC-IV), *PRI* Perceptual Reasoning Index WISC-IV, *SSP* Short Sensory Profile

#### ASD cohort

We included 2377 families (male, *n* = 2049) with ASD from the Simons Simplex Collection (SSC) [22] including 1786 quads and 591 trios. The SSC is overseen by SFARI (Simons Foundation Autism Research Initiative) in collaboration with 12 university-affiliated research clinics. Parents consented and children assented as required by each local institutional review board. Participants were de-identified before data distribution. This resource includes individuals (confirmed to have ASD) and their nuclear family members, with recruitment limited to families in which only a single individual has met research criteria for ASD, including first cousins. The nuclear family also includes an unaffected sibling. Families were excluded if there was intellectual disability or schizophrenia in a sibling or parent. Each proband was evaluated with a detailed battery of assessments including the ADOS; [23])and the Autism Diagnostic Interview-Revised (ADI-R; [24]).

### Description of biologic materials

#### Sample collection

Upon consent to participate in this study, families were directed to the UCSF pediatric phlebotomy lab or a local lab of their choice to obtain ~ 8 ml of whole blood in an ACD tube for processing by the UCSF Genome Core Facility.

#### DNA preparation

DNA was isolated using the Qiagen Gentra Puregene system. DNA quality was confirmed by standard 260/280 ratios and agarose gel visual inspection. Prior to library generation and exome sequencing, DNA was tested for purity and size using the Agilent Bioanalyzer.

#### WES

The DNA was fragmented using a Covaris E220 ultra sonicator to a size range of 350-450 bases. After fragmentation, the DNA was processed using the Agilent library preparation kit following the manufacturer’s protocol. Exome sequencing was performed using the Nimblegen Human SeqCap EZ Exome (v3.0) kit according to the manufacturer’s protocol. This kit targets genes from CCDS.2, Vega, Gencode and Ensembl in addition to microRNA’s from miRBase and snoRNABase, for a total of over 20,000 genes and 64 Mb of covered genomic region. This yields an average of > 60× coverage overall for the sequenced bases.

#### Rare single nucleotide variants (rSNV) analytic pipeline

Our variant analysis follows ‘The Broad Institute’s Best Practices’ guidelines for discovering putative variants and utilizes the Genome Analysis Toolkit (GATK; software version 2014.2-3.1.7-10) in combination with BWA-mem, Picard Tools, and SAM Tools [25–28]. After aligning the DNA read sequences to the GRCh37 reference build using BWA-mem, Picard Tools is used to identify and remove PCR duplicates, add read group information, and sort alignment files using modules Mark Duplicates, SortSam, and AddOrReplaceReadGroups respectively. Subsequently, GATK modules RealignerTarget Creator is used to identify putative indels and IndelRealigner is used to realign around those intervals. Base recalibration is performed using the GATK modules BaseRecalibrator in combination with PrintReads to produce sample specific BAM files. Variant calling is performed using GATK HaplotypeCaller in combination with CombineGVCFs module to produce sample specific gVCF files. These individual patient/parent files are combined, annotated, and genotyped over intervals of interest using GenotypeGVCFs to produce a single project specific VCF file of variants. GATK modules, VariantRecalibrator and ApplyRecalibration are used to add a VQSLOD score (confidence score that estimates the probability that the variant is a true positive) using HapMap 3.3, the Omni 2.5 SNP BeadChip, 1000 Genome, and Mills indels as training sets.

The resulting VCF file is then stored in a MySQL database table with separate rows for each variant and columns representing VCF file format required headers. Each variant in the database is annotated against a reference transcript. For variants falling within coding regions, codon affect is assessed with nonsynonymous identified variants further analyzed using Polyphen-2 for predictive damage. All variants are cross-referenced against public and private datasets to assess population frequency. These include data from the Exome Sequencing Project and 1000 Genomes. Variants are further annotated against UCSC genome tracks as well as external location specific or gene specific datasets. The resulting relational database permits complex initial filtering of variants by protein consequence (synonymous, nonsynonymous, stop, and frameshifts), location (within gene boundaries, exon, boundaries, and splice sites), and confidence score (VQSLOD, polyphen, SIFT, RVIS). Once a subset of variants is identified, sample genotype information can be processed to assess inheritance pattern.

### Determination of variant significance

For de novo analysis, variants were required to be missense, indel, or within 3 base pairs of a splice site, have a VQSLOD score greater than 0, and be below a population frequency of 1% (as determined by 1000 Genomes and the Exome Variant Server). In addition, affected genotype quality (GQ) should be greater than 85 and have a minimum of 10 reads with at least 3 showing the alternate variant. Both parents are required to have a GQ greater than 50 and no more than 3 reads showing the alternate variant. For inheritance analysis given computational limitations, we limited our scope to missense variants within ASD or the Coronary Artery Disease (CAD) comparision genes which had a VQSLOD greater than 2 and a population frequency below 1% [29]. Gene lists are included in Additional files 1 and 2. For each gene group, variants were separated into subgroups of transmitted (passed from parent to child) or non-transmitted (not passed from parent to child).

#### Variant confirmation

De novo variants were first directly examined by inspection of the aligned reads in the proband and both parents using the integrated genomics viewer (IGV). Sanger sequencing using well-established approaches confirmed candidates that remained after this inspection.

### Statistical analysis

#### De novo analysis

All variants were compared to parental samples to determine if they were de novo or inherited from the biological mother or father. We investigated the biological relevance of the affected genes based on human and animal literature reported in the Online Mendelian Inheritance in Man database.

#### Enrichment analysis

Statistical analysis of enrichment for transmitted and non-transmitted rSNV was conducted using a set of 76 high probability candidate genes linked to ASD from SFARI Gene 2.0 (AutDB) [30]. These high-risk genes were determined using the SFARI Gene database. This database utilizes a human curated biological approach, linking information on autism candidate genes within its original Human Gene Module to corresponding data within diverse modules such as Animal Model, Protein Interaction, Gene Scoring, and Copy Number Variant. Each ASD risk gene is classified in a specific category using a set of annotation rules developed by an advisory board. Seventy-six genes from AutDB (date pull 05.21.15) were determined to be “probably damaging” and were included in the high-risk ASD gene set whereas 292 were categorized as possibly damaging and included as moderate-risk ASD gene set (see Additional files 1 and 2.)

Assuming random draws from the genome, the probability of drawing a mutation from the gene set can be calculated as the sum of transcript lengths in the gene set divided by the total length of the assayed transcriptome. To determine the probability that each individual exhibits a mutation enrichment in a designated gene set (e.g., high-risk autism gene set or moderate-risk autism gene set), the number of gene set-specific mutations was compared to the expected distribution as modeled by the binomial distribution and parameterized by the length-corrected draw probability described above. The resulting probability describes the gene set enrichment score for that individual. To obtain a population level probability, each individual probability was converted into a z-score equivalent, and a Chi Square test was performed with a number of degrees of freedom equal to the number of individuals in the cohort - 1. This overall Chi Square probability describes the population enrichment of mutations in the relevant set of genes. For the ASD/CAD analysis of SSC samples, the analysis was performed as described above, except for computational reasons related to processing data for 8917 samples, a list of 580 CAD genes were used as the background instead of the entire genome.

## Results

### De novo mutation analysis

We conducted WES in 11 SPD trios. Given the limitation of power with this sample size, we have chosen to conduct this initial analysis by focusing on de novo loss of function and missense mutations. We identified 12 candidate genes with de novo loss of function and/or missense variants in our 11 SPD probands. Among these genes, there were two (18%) de novo mutations (one each, nonsense and missense) in neurodevelopment candidate genes: MBD5 and FMN2. The nonsense mutation in MBD5 leads to a premature termination of the protein at serine 318 (S318X). The missense in FMN2 leads to a proline to leucine amino acid substitution (P927L), which is predicted to have a damaging effect. Based on standards and guidelines for interpretation of sequence variants, the MBD5 de novo loss of function mutation would be considered pathogenic with a very strong evidence of pathogenicity [31]. The FMN2 is also predicted to be pathogenic- however given that it is reported in the ExAC database, the formal clinical interpretation would be a “variant of unclear significance.” Nine additional mutations were identified (Table 2). The changes in MBD5, FMN2, DNAH9, KLHL33, MCM2, PFDN6, and SLCO2B1 were confirmed by Sanger sequencing.

**Table 2:** De novo variance in children with SPD

	PolyPhen2 HVAR score	Mutation type	AA position	AA change	Chromosome position	Base change
MBD5	–	Stop	318	S- > Stop	149,226,465	TCA - > TAA
FMN2	.86	Missense	947	P- > L	240,370,952	CCT - > CTT
DNAH9	.88	Missense	2716	R- > W	11,696,904	CGG - > TGG
KLHL33	.96	Missense	263	R- > W	20,898,048	CGG - > TGG
PFDN6	.99	Missense	62	P- > L	33,258,152	CCG - > CTG
SLCO2B1	1.0	Missense	651	L- > P	74,915,513	CTG - > CCG
MCM2	0	Missense	636	P- > L	127,337,968	CCG - > CTG
TULP4	.06	Missense	1456	G- > R	158,925,061	GGG - > AGG
SPTYD1	.003	Missense	135	N- > S	18,637,417	AAT - > AGT

*AA* Amino acid, *S* Serine, *P* Proline, *L* Leucine, *R* Arginine, *W* Tryptophan, *G* Glycine, *N* Asparagine

### Enrichment of rare single nucleotide variants in ASD associated genes

#### Experiment 1: Burden of rSNV in children with SPD and their parents

Given the literature suggesting strong heritability of sensory over-responsivity and the co-occurrence of sensory processing dysfunction in autism, we sought to determine whether there was a greater than chance inheritance of rSNV from amongst the high and moderate risk ASD candidate genes. We found that the children with SPD show trend level enrichment of inherited high risk ASD rSNV (*p* < 0.068, approximately 1/14 chance of false positive) with all individual children showing the same direction of rSNV burden (i.e. each child inherited greater than 50% of the available deleterious rare alleles in the high probability ASD genes) for this gene set. By contrast, these 11 children did not show an increase burden of variants in the moderate-risk ASD gene set (*p* = 0.966).

**Fig. 1:**
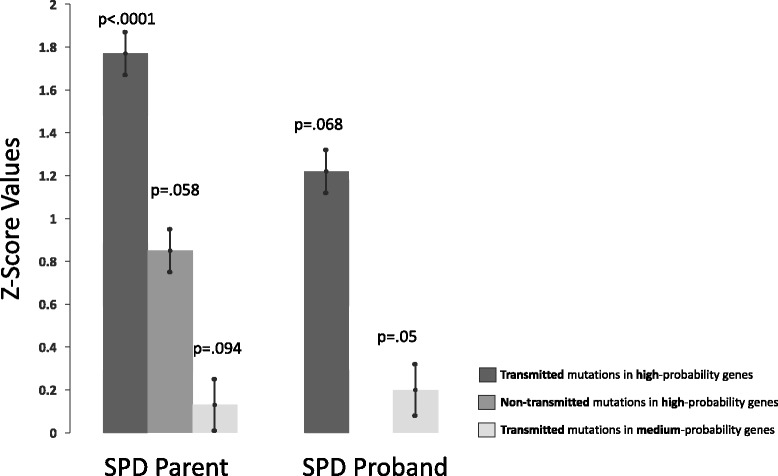
Enhanced burden of rSNV in children with SPD and their parents

Based on the increased burden of inherited rSNV in high risk ASD genes in children with SPD, we sought to explore the burden of variants from amongst the high- and moderate- risk ASD genes in their parents—including variants that were passed to their affected children (transmitted) and those that were not (non-transmitted). The data suggests that parents of children with SPD have a significant enrichment of transmitted variants in the high-risk ASD genes (*p* = < 2.4e-10) which exceeds the association for non-transmitted high probability ASD genes (*p* = < 0.058) or transmitted moderate-risk ASD genes (*p* < 0.942; Fig. 1.)

#### Experiment 2: Burden of rSNV in children with ASD, their parents and unaffected siblings

**Fig. 2:**
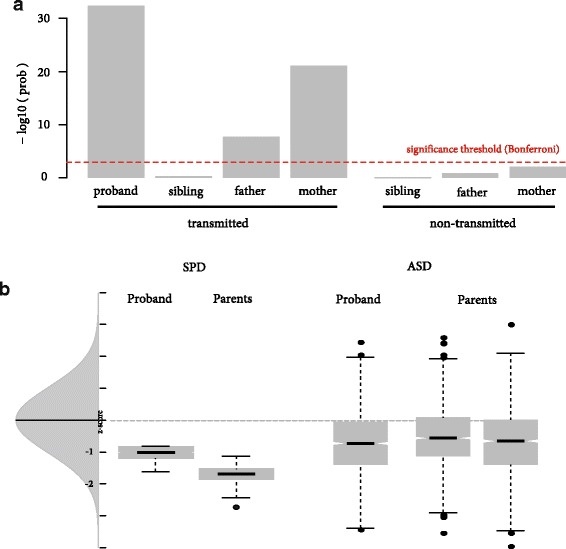
**a**. Enrichment of transmitted and non-transmitted rSNV in ASD simplex families. Population significance testing (ASD mutations / Coronary Artery Disease (CAD) mutations) assessing burden of inherited/transmitted variants in high-risk candidate ASD genes relative to coronary artery disease genes. Parents and siblings do are non-ASD. **b** Enrichment of rSNV in SPD and ASD cohorts. Probability analysis comparing rSNV enrichment in SPD cohort to the ASD population

Based on finding an enriched burden of inherited rare genetic variants, specifically in the high- but not moderate-risk ASD genes in our small SPD cohort, we aimed to determine if this finding was also evident in a large simplex cohort of children with ASD and their parents. In a group of children with ASD, with parents and siblings who do not meet criteria for ASD, we found that the parents and the affected child with ASD show an enhanced burden of inherited/transmitted variants in high-risk candidate ASD genes relative to genes from an unrelated condition, coronary artery disease. By contrast, the unaffected siblings do not show this increase in high-risk candidate ASD genes variants that were transmitted to the proband. Additionally, unaffected parents and siblings do not show an increase in the number of variants in non-transmitted high-risk candidate ASD genes (Fig. 2a). In comparison with the small SPD cohort, the large SSC ASD cohort shows a greater range of variant burden than the SPD cohort. Finally, the SPD cohort has equivalent or greater burden of genetic variants in high-risk ASD genes relative to the ASD group (Fig. 2b).

## Discussion

There is growing interest in the etiology of sensory processing dysfunction for individuals with social communication challenges meeting DSM-5 criteria for ASD. This stems in part from the fact that in the current version of the DSM, “hyper- or hyporeactivity to sensory input or unusual interests in sensory aspects of the environment” is now included in the ASD phenotypic criteria. There are, however, many individuals who are over-responsive to sensory input but do not have the degree of social or communication challenges that meet an ASD label. These individuals are currently being lumped under the category of Sensory Processing Disorder or SPD. With an estimated 10–15% of children with an ASD label currently being reported to have disease associated variants identified via WES, we sought to investigate the occurrence of de novo missense/nonsense mutations in ASD candidate genes in children with SPD. Furthermore, we sought to investigate whether children with SPD and their parents would show an increased burden of deleterious rSNV in high and moderate-risk ASD candidate genes. Herein, we report that 18% of our sample has a pathogenic de novo missense/nonsense mutation in genes previously associated with neurodevelopmental disorders. We further show that there is an enhanced rate of rare inherited variants in high-risk ASD genes transmitted from parent to affected child in SPD and ASD patients, but not in unaffected ASD siblings.

In this report, WES has identified a stop codon mutation in MBD5 that likely causes premature truncation and nonsense mediated decay of the protein from one of the two alleles leading to haploinsufficiency. Methyl-CpG-binding domain 5 (MBD5) is a gene located at 2q23.1. This gene, reviewed by Mullegama, et al. 2016, is believed to contribute to DNA methylation and through that to potentially be involved in cell division, growth, and differentiation however further research is indicated to better understand the role of this protein [32]. Haploinsufficiency is believed to impact the expression of downstream genes such as upregulation of CF4 and UBE3A, and down regulation of MEF2C, EHMT1, RAI1 in a dose sensitive fashion [33]. Loss of function mutations in MBD5 are highly likely to be pathogenic given that the probability of loss of function intolerance is 1.00 [34].

MBD5, also referred to as mental retardation autosomal dominant 1 and now given the name MBD5-Associated Neurodevelopmental Disorder (MAND), was originally described in the context of the 2q23.1 microdeletion syndrome thought to be an Angelman Syndrome mimic. Clinically, affected individuals are variably affected by intellectual disability, motor delay, and severe speech impairment. The language deficits, social challenges and stereotypies seen in affected individuals can result in the affected child meeting criteria for autism [35, 36]. Additionally, children may have seizures, sleep disorders, and attentional challenge and some individuals will show mild craniofacial and skeletal anomalies [33, 37]. In the review article by Hodge et al. 2014, they summarize the phenotype for individuals in the literature with either point mutations in the MBD5 gene or microdeletions containing MBD5. In their summary table, they include reports of sensory integration disorder (SID) which is a label frequently used in the occupational therapy community. In children with MBD5 point mutations, there is no comment on SID, however SID is reported in 2/3 (66%) children with 2q23.1 microdeletions [33]. So while, sensory over-responsivity to either sound or touch was a key clinical inclusion phenotype in our cohort, it is not always considered in the current genetic literature and thus difficult to know the extent to which it affects children with single gene disorders.

The female patient in this study first presented to our SNAP clinic at age 11 years due to prominent sensory dysfunction affecting her auditory, vestibular, visual, tactile, and oral systems (classified as 2 standard deviations below average on the Sensory Profile [38]). On evaluation by a pediatric geneticist, no dysmorphic features were noted and she is normocephalic with a head circumference at the 44th percentile. On evaluation by a licensed community pediatric neuropsychologist (L.D.) using the Wechsler Intelligence Scale for Children-4th edition (WISC IV), she had a verbal comprehension index of 121, Perceptual Reasoning Index of 86, and a working memory index of 99. These scores are all in the average to above average range but highlight a relative challenge in the perceptual measures. While verbal conceptual skills are a strength, multiple aspects of motor control are a significant concern with poor articulation and dysgraphia leading to severe school based challenges. In addition, while socially alert, interested and driven, she has challenges with interpretation of non-verbal social cues as well as heightened distractibility affecting sustained focus and selective attention. Specifically, she meets cut-off criteria for combined Inattentive/Hyperactive subtype on the Vanderbilt ADHD Diagnostic Parent Rating Scale [39]. These challenges contribute to a lack of social finesse that has led to difficulty with maintaining age appropriate friendships. However on evaluation in the lab, in the community, and at school, she did not meet the social communication criteria for an autism spectrum disorder.

Of the nine genes with missense mutation leading to amino acid substitution, six were considered potentially damaging to the protein structure: FMN2, DNAH9, KLHL33, MCM2, PFDN6, SLCO2B1. Of these genes, only Formin 2 (FMN2) has been previously reported to be associated with neurodevelopmental impairment. FMN2, located at chromosome 1q43, is one of 15 members of the formin homology protein family and is thought to play a role in actin cytoskeleton organization and cellular polarity. FMN2 has received the designation: mental retardation, autosomal recessive 47. In addition to the literature implicating mutations of FMN2 with autosomal recessive inheritance, there are reports of heterozygous deletion involving FMN2 in two additional reports in patients with neurodevelopmental impairment [40, 41]. Law, et al. 2014 reports that FMN2 localizes to the dendrites and likely alters synaptic density in a mouse model that has demonstrated challenges with fear-learning [42]. Affected patients were reported to have challenges with cognition and speech out of proportion to their motor difficulties. In the existing literature, there is no report of associated dysmorphic features and in one family there were rare complex partial seizures. There is no mention of sensory processing ability or challenges in the extant literature.

The male patient in this study with the FMN2 missense mutation first presented to SNAP research at 9 years of age. The patient had sensory dysfunction affecting his auditory, vestibular, tactile, multisensory and oral systems (classified as 2 standard deviations below average on the Sensory Profile [38]). The patient had a verbal comprehension index of 116, Perceptual Reasoning Index of 115, and a working memory index of 94 as assed using the WISC IV. He did not meet ASD cut-off scoring using the ADOS Assessment. Finding these two, highly penetrant de novo mutations in SPD patients who do not meet criteria for ASD suggests that de novo mutations may be found as the primary etiology in a significant percentage (up to 18%) of children with a sensory-first presentation.

While it has long been recognized that triplet repeat and single gene disorders, such as Fragile X or SHANK2, and more recently copy number variation disorders, such as 16p11.2 deletion, are associated with neurodevelopmental conditions; there has also been substantial interest in whether a cumulative burden of rare single nucleotide variants, either inherited or de novo, can result in a clinical condition such as ASD or SPD. In this study, we looked first at our SPD pediatric cohort with the hypothesis that these affected children would have a higher burden of inherited rSNV in high- or moderate-risk ASD genes relative to the expected mutation rate. We found that, despite the small number of individuals in this pilot SPD cohort, there was indeed a trend level increase in transmitted rSNV in high-risk ASD candidate genes but not moderate-risk ASD genes. This result has two main implications. This finding suggests, first, that the SPD phenotype and the ASD phenotype may have shared genetic underpinnings in a “high value” gene set, as of now only 76 genes. Second, the phenotype may also result from an accumulation of multiple changes each with a smaller effect size, hence polygenic (and thus inherited from parents). Given that in the ASD literature, parents have been reported to show an increase in sensory processing behavioral differences, we investigated the burden of rSNV in the parents of our probands with SPD [43, 44].

Despite the small number of individuals in the cohort, there was a robust increase in the transmitted high risk ASD gene rSNV for parents of children with SPD. There was no increase in rSNV for either transmitted or non-transmitted moderate-risk ASD genes. This finding supports the importance of investigating the role of variant burden in SPD with a large effect in a small sample, and also highlights a potential difference in causality between the high and moderate-risk candidate genes. This robust increase in burden of variants in the parents is interesting given that the ASD literature suggests increased sensory differences in parents of affected individuals. In this SPD cohort, there are a couple of explanations that merit further exploration. First, it is possible that SPD parents themselves are affected by sensory processing dysfunction that is similar to their children’s. Second, one must consider that the probands have additional variants contributing to their clinical symptomatology that we are not measuring in the exonic DNA meriting a whole genome approach. Finally, it may not be simply the additive burden of the variants but rather a particular combination of variants in specific genes that may work in an epistatic fashion to contribute to sensory processing dysfunction.

We chose to further investigate the relationship between rSNV burden in affected children with SPD and their parents by applying this analysis to a cohort of children also known to have an increased prevalence of sensory difference, those with ASD. In the SSC ASD family cohort, we were also able to increase our statistical power both by the sheer number of ASD families and by the inclusion of an unaffected sibling in the family cluster. We thus investigated whether children with ASD from the SSC and their unaffected siblings and parents have a higher burden of rSNV that were transmitted to the identified proband with ASD. As predicted, the affected child when compared to his or her parents, but not the unaffected sibling, shows an increase in transmitted rSNV from the high-risk ASD gene set. After stringent Bonferroni correction, neither the unaffected siblings nor the parents showed a significant burden of non-transmitted rSNV. These findings underscore the importance of inherited variants in ASD, even in families recruited for an increased likelihood of de novo variants. In one recent genome-wide ASD study, children with ASD show a “nominal difference” in rare inherited nonsense/splice site mutations when compared to their unaffected siblings [45]. Similarly, a study including 3871 ASD cases investigating the interplay of common and rare variants, reports that 5% of the ASD cohort has de novo loss of function mutation in a set of 107 autosomal genes involved in synaptic formation, transcriptional regulation, and chromatin remodeling pathways. However, this study did not show an association for inherited missense variants, so these variants were not included in their Transmission And De novo Associated (TADA) analysis [46]. De Rubeis et al., 2014 suggest that while the de novo loss of function (LOF) mutations confer the largest effect on risk, by including de novo missense SNV and transmitted LOF variants, they were able to double their gene discovery rate and suggest that ASD genes show “a strong constraint against variation.” Future genetic investigation to determine whether there are genes specific to sensory challenges, specifically SOR, and genes more specific to language and social differences would greatly contribute to our understanding of neurodevelopment and neurodevelopmental disorders.

There are limitations to this work, which bear mentioning. This work needs to be replicated in a much larger independent sample, despite the provocative findings in this initial cohort. In future investigations, direct assessment of auditory and tactile SOR phenotype in parents and children with isolated SPD and ASD/SPD is warranted. Finally, bringing direct sensory phenotyping in a broader cohort of children with neurodevelopmental concerns with their parents and siblings in conjunction with genetic investigation will deepen our understanding of the contributing genetic variations, both monogenic and polygenic.

## Conclusions

In this study, we find a rough estimate of 18% de novo, disease-associated, single gene mutations in a cohort of children identified on the basis of a sensory processing dysfunction. Furthermore, we find that this pediatric sensory cohort and their parents, similar to an autism cohort, show an enrichment of rare single nucleotide variants in genes previously reported to be associated with autism and neurodevelopmental delay. This small-scale study suggests that SPD results from genetically coded, brain based differences, with monogenic and polygenic contributions. It highlights the need for additional genetic research in SPD and the importance of a thorough genetic evaluation in children presenting with sensory processing dysfunctions, regardless of the additional neurodevelopmental concerns.

## Additional files

Additional file 1: High- Probability Risk ASD Gene Set. Table of seventy-six genes from AutDB determined to be “probably damaging” and included in the high-risk ASD gene set. (XLS 80 kb)

Additional file 2: Moderate- Probability Risk ASD Gene Set. Table of 292 genes from AutDB categorized as possibly damaging and included as moderate-risk ASD gene set. (XLS 151 kb)

## Abbreviations

ADOS: Autism Diagnostic Observation Schedule
ASD: Autism Spectrum Disorder
CAD: Coronary Artery Disease
DSM: Diagnostic and Statistical Manual
FMN2: Formin 2
GATK: Genome Analysis Toolkit
GQ: Genotype quality
ID: Intellectual disability
LOF: Loss of function
MBD-5: Methyl-CpG-binding domain 5
rSNV: Rare Single Nucleotide Variants
SCQ: Social Communication Questionnaire
SFARI: Simons Foundation Autism Research Initiative
SNAP: Sensory Neurodevelopment and Autism Program
SNV: Single Nucleotide Variants
SPD: Sensory processing dysfunction
SSC: Simons Simplex Collection
UCSF: University of California, San Francisco
WES: Whole exome sequencing
